# A Transient Homotypic Interaction Model for the Influenza A Virus NS1 Protein Effector Domain

**DOI:** 10.1371/journal.pone.0017946

**Published:** 2011-03-28

**Authors:** Philip S. Kerry, Juan Ayllon, Margaret A. Taylor, Claudia Hass, Andrew Lewis, Adolfo García-Sastre, Richard E. Randall, Benjamin G. Hale, Rupert J. Russell

**Affiliations:** 1 Biomedical Sciences Research Complex, University of St. Andrews, St. Andrews, Fife, United Kingdom; 2 Department of Microbiology, Mount Sinai School of Medicine, New York, New York, United States of America; 3 Division of Infectious Diseases, Department of Medicine, Mount Sinai School of Medicine, New York, New York, United States of America; 4 Global Health and Emerging Pathogens Institute, Mount Sinai School of Medicine, New York, New York, United States of America; University of Cambridge, United Kingdom

## Abstract

Influenza A virus NS1 protein is a multifunctional virulence factor consisting of an RNA binding domain (RBD), a short linker, an effector domain (ED), and a C-terminal ‘tail’. Although poorly understood, NS1 multimerization may autoregulate its actions. While RBD dimerization seems functionally conserved, two possible apo ED dimers have been proposed (helix-helix and strand-strand). Here, we analyze all available RBD, ED, and full-length NS1 structures, including four novel crystal structures obtained using EDs from divergent human and avian viruses, as well as two forms of a monomeric ED mutant. The data reveal the helix-helix interface as the only strictly conserved ED homodimeric contact. Furthermore, a mutant NS1 unable to form the helix-helix dimer is compromised in its ability to bind dsRNA efficiently, implying that ED multimerization influences RBD activity. Our bioinformatical work also suggests that the helix-helix interface is variable and transient, thereby allowing two ED monomers to twist relative to one another and possibly separate. In this regard, we found a mAb that recognizes NS1 via a residue completely buried within the ED helix-helix interface, and which may help highlight potential different conformational populations of NS1 (putatively termed ‘helix-closed’ and ‘helix-open’) in virus-infected cells. ‘Helix-closed’ conformations appear to enhance dsRNA binding, and ‘helix-open’ conformations allow otherwise inaccessible interactions with host factors. Our data support a new model of NS1 regulation in which the RBD remains dimeric throughout infection, while the ED switches between several quaternary states in order to expand its functional space. Such a concept may be applicable to other small multifunctional proteins.

## Introduction

During infection the influenza A virus NS1 protein participates in multiple protein-RNA and protein-protein interactions to perform a plethora of functions (reviewed in [1]). Examples include its ability to act as a potent interferon (IFN) antagonist (both pre- and post- transcriptionally) [2], [3], [4], [5], its inhibition of host antiviral enzymes [6], [7], its enhancing effect on viral translation [8], and its activation of phosphoinositide 3-kinase (PI3K) signaling [9].

Approximately 30 cellular and viral factors have been reported to interact either directly or indirectly with NS1, which seems surprising given that NS1 itself is relatively short (only ∼230 amino-acids). However, protein multifunctionality may be a key feature of many small RNA virus replication strategies given that they usually possess a restricted coding capacity. Potential mechanisms that likely influence the functions of NS1 include: (i) post-translational modifications [10], [11], [12]; (ii) strain-specific polymorphisms [13], [14], [15], [16], [17], [18]; and (iii) spatio-temporal distribution [19], [20]. As with many cellular proteins, different multimeric forms may also be an important determinant of specific NS1 functions [21], [22].

**Figure 1:**
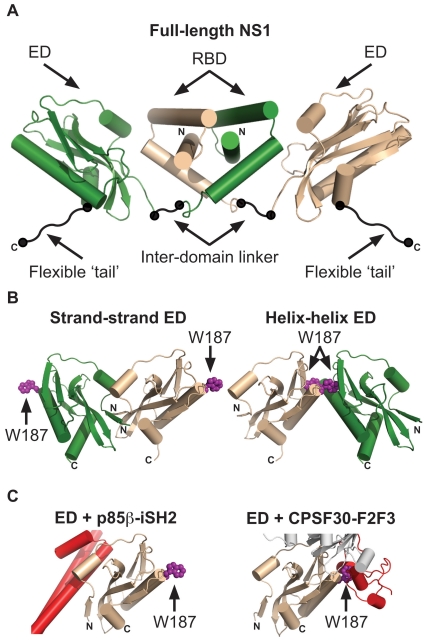
Structures of the influenza A virus NS1 protein. (**A**) **Full-length dimeric NS1.** The RNA-binding domain (RBD), effector domain (ED), inter-domain linker, and flexible ‘tail’ are labeled. Neither the inter-domain linker nor the flexible ‘tail’ have been observed in crystal structures, and are therefore represented schematically. Figure generated using PDB ID 3F5T. (**B**) **The two proposed NS1 ED dimerization interfaces: strand-strand and helix-helix.** W187 is highlighted in both structures. Figure generated using PDB ID 2GX9. (**C**) **Incompatibilities between ED homodimerization and ED binding cellular proteins.** Shown are complexes of NS1 ED with the p85β-iSH2 domain (left panel, PDB ID 3L4Q) and CPSF30-F2F3 domain (right panel, PDB ID 2RHK). W187 is highlighted for reference. Note incompatibilities between NS1:p85β, the strand-strand dimer (**Fig. 1B, left**), and the full-length NS1 dimer (**Fig. 1A**). Note incompatibilities between NS1:CPSF30 and the helix-helix dimer (**Fig. 1B, right**). In all panels, NS1 monomers are colored green or wheat, and cellular proteins are colored red. The N- and C- termini of NS1 constructs are labeled. For the NS1:CPSF30 structure in **Fig. 1C, right** the second monomers of NS1 ED and CPSF30-F2F3 that make up the published tetrameric complex [33] are shown in gray.

The N-terminal 73 amino-acid residues of NS1 form a symmetrical homodimeric RNA-binding domain (RBD) [23], [24], [25], [26], which is connected to the central effector domain (ED; residues 86-204) via an inter-domain linker [27]. The final ∼25 residues of NS1 appear to be unstructured, and are termed the C-terminal flexible ‘tail’ (FT) (**Fig. 1A**) [28]. Both the isolated RBD and ED can homodimerize in solution [26], [28], [29], [30], and both contribute to functional NS1 multimerization during infection [21], [22]. Surprisingly, the recent full-length structure of NS1 revealed that the EDs do not contribute to the overall dimer interface, but instead flank the core dimeric RBDs, thus creating a ‘domain-swapped’ dimer (**Fig. 1A**) [27]. Nevertheless, within the crystal lattice, the EDs formed homotypic contacts with neighboring molecules, thereby confirming that higher-order oligomeric forms of NS1 may occur [27].

The isolated NS1 RBDs of several different influenza A virus isolates all show a very high degree of structural homology and conservation (refs. [24], [26] and PDB IDs 2Z0A & 3M8A). In particular, the mode of RBD dimerization does not vary between strains, and is unaffected by binding double-stranded RNA (dsRNA) [25]. In contrast, several possible ED homodimer interfaces have been proposed based upon crystal structures obtained using NS1 proteins from various strains. Initially, an interface mediated by short β-sheets (strand-strand dimer; residues 88-91) of two NS1 molecules was suggested from the structure of the NS1 ED from mouse-adapted A/Puerto Rico/8/34 (PR8) (**Fig. 1B**) [29]. However, later structures of EDs from A/Duck/Alberta/60/76 (Alb/76) [28] and A/Udorn/72 (Ud/72) [30] did not possess this interface; instead dimerization was mediated by homotypic interactions between the long α-helices (residues 170–188) of each ED (helix-helix dimer; **Fig. 1B**). Of note, this helix-helix interface was also present within the original PR8 ED structure as an alternative to the strand-strand dimer, but was not deemed relevant based on its small dimerization interface [29]. Nevertheless, mutation of W187, a residue responsible for key reciprocal hydrophobic interactions at the helix-helix interface (**Fig. 1B**), caused the isolated Alb/76 and Ud/72 EDs to behave as monomers, thus providing direct experimental evidence that the helix-helix ED dimer predominates in solution [28], [31].

The precise multimeric state of functional NS1 in a biological context is far from clear. Indeed, there is already circumstantial evidence from structures of the NS1 ED in complex with different cellular binding partners that suggests this domain cannot adopt either strand-strand or helix-helix states when carrying out certain functions (**Fig. 1C**) [32], [33]. In this study we sought to assess the relevance of different ED homodimer interfaces using both biophysical and bioinformatical techniques. Crystallographic uncertainties over protein-protein interfaces have often been resolved by observing the same interface in homologous proteins [34]. To this end, we present several new crystal structure forms of different wild-type dimeric and rationally-designed monomeric NS1 EDs grown under novel conditions, and confirm that the helix-helix ED dimer represents the major dimerization interface for this protein. We also analyze the properties of this weak, transient interface using bioinformatics, functional biochemical assays and a newly characterized monoclonal antibody that is unlikely to recognize the helix-helix dimeric ED.

## Results

### The helix-helix dimer interface is conserved among all NS1 ED structures

The favored dimerization interface of the NS1 ED remains controversial. To date, the isolated EDs of four influenza A virus strains have been examined by x-ray crystallography, giving rise to two proposed homodimer forms: strand-strand and helix-helix ([28], [29], [30] and PDB ID 3M5R) (**Fig. 1B**). In order to examine the possible dimerization arrangements of this domain in more detail, EDs of the divergent mouse-adapted human influenza A virus PR8 and avian influenza A virus Alb/76 were expressed and purified from bacteria. Consistent with previous reports, both EDs were dimeric in solution as determined by gel filtration (*data not shown* and [28]). The purified EDs were screened for new conditions under which crystallization may occur in addition to those previously characterized. Four novel crystal forms were identified (three for PR8 NS1 ED and one for Alb/76 NS1 ED) and solved by x-ray crystallography using molecular replacement (**Table 1**). Three of the ED structures contained two molecules of NS1 in the asymmetric unit (PDB IDs 3OA9, 3O9S, and 3O9T), whereas eight monomers were present in the other structure (PDB ID 3O9U) (**Table 2**).

**Table 1:** Data collection and refinement statistics for NS1 effector domains.

PDB ID	3O9S	3O9T	3O9U	3OA9	3O9Q	3O9R
Protein	PR8 ED	PR8 ED	PR8 ED	Alb/76 ED	PR8 ED (W187A)	PR8 ED (W187A)
Space group	P2_1_2_1_2_1_	C222_1_	P6_4_	P6_1_	P3_2_21	P2_1_2_1_2_1_
Cell dimensions (Å)	a = 48.71 b = 57.02 c = 101.43	a = 72.40 b = 114.87 c = 106.42	a = 114.12 b = 114.12 c = 199.29	a = 47.95, b = 47.95, c = 231.54	a = 67.52, b = 67.52, c = 158.98	a = 33.60, b = 70.11, c = 104.26
Resolution (Å)*	2.50 (2.54–2.50)	2.20 (2.24–2.20)	3.20 (3.31–3.20)	2.90 (2.95–2.90)	2.50 (2.54–2.50)	2.00 (2.03–2.00)
R_sym_ (%)*	8.5 (41.6)	4.2 (48.4)	10.2 (59.3)	12.4 (55.3)	4.9 (47.2)	6.2 (32.1)
*I/*σ*I* *	28.5 (4.3)	30.0 (2.9)	14.8 (2.8)	19.3 (2.8)	43.7 (3.4)	28.7 (2.7)
Completeness (%)*	92.4 (77.0)	96.4 (80.9)	97.2 (80.4)	99.2 (90.6)	99.4 (99.0)	95.5 (63.5)
Unique reflections	9692	22031	23562	6694	15144	16609
Redundancy	6.3	3.3	4.2	4.9	6.0	3.5
R_work_ (%)	19.6	24.2	17.5	22.0	24.7	19.6
R_free_ (%)	25.7	27.0	22.2	22.8	30.2	25.3
Protein atoms	1895	1869	7247	1791	1885	1836
Water atoms	102	28	0	16	30	206
Rmsd bonds (Å)	0.008	0.026	0.008	0.01	0.008	0.008
Rmsd angles (°)	1.126	2.004	1.165	1.344	1.082	1.048
Average Bfactor	36.0	42.6	85.8	41.2	66.0	30.6

Structures of PR8 ED-WT, PR8 ED-W187A, and Alb/76 ED-WT were solved by molecular replacement using existing PR8 and Alb/76 structures (PDB IDs 2GX9 and 3D6R).

*Values in parentheses are for highest resolution shell.

**Table 2:** Occurrence of proposed dimeric arrangements in NS1 ED structures solved to date.

Strain	Spacegroup	PDB ID	Monomers in asymmetric unit	Helix-Helix	Strand-Strand
PR8	P4_3_22	2GX9	2	4	4
PR8	P6_4_	3O9U*	8	4	4
PR8	P2_1_2_1_2_1_	3O9S*	2	4	8
PR8	C222_1_	3O9T*	2	4	8
Alb/76	P6_5_22	3D6R	2	4	8
Alb/76	P6_1_	3OA9*	2	4	8
Ud/72	C222_1_	3EE8	2	4	8
Ud/72	P2_1_2_1_2_1_	3EE9	2	4	8
Cal/09	P2_1_	3M5R	6	4	8

New crystal structures reported here are marked with an asterisk.

**Figure 2:**
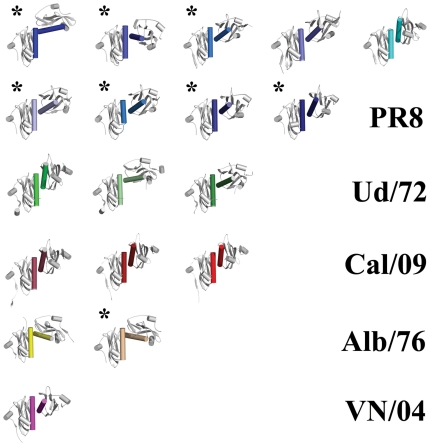
The helix-helix ED dimer is present in all known apo ED crystal structures. All observed ED helix-helix dimers oriented via the left-hand monomer of each pair show the wide range of positions possible for the right-hand monomer. NS1 ED helix-helix dimers arranged as follows (identified by PDB ID_chains): (top row, from left to right) 3O9T_AA, 3O9T_BB, 3O9S_AB, 2GX9_AB, 3L4Q_AC; (second row) 3O9U_AC, 3O9U_BE, 3O9U_DG, 3O9U_FH; (third row) 3EE9_AB, 3EE8_AA, 3EE8_BB; (fourth row) 3M5R_AB, 3M5R_DE, 3M5R_FG; (fifth row) 3D6R_AB, 3OA9_AB; (bottom row) 3F5T_AA. New crystal structures reported here are marked with an asterisk. Note that dimers 3O9T_AA, 3L4Q_AC and 3F5T_AA are excluded from bioinformatic analyses (see Table 3) as they are either in complex with another molecule or are part of a full-length NS1 structure.

Comparison of these new structures with previously published structures of the PR8 ED and Alb/76 ED indicated that in all cases contacts are formed at the helix-helix dimer interface, either within the asymmetric unit (3OA9, 3O9U, and 3O9S) or on a 2-fold crystallographic axis (3O9T). In contrast, the strand-strand dimer interface is totally absent except for 3O9U (**Table 2**). Extension of these comparisons to all reported isolated NS1 ED structures indicates that the helix-helix dimer is ubiquitous, suggesting that the contacts at this dimer interface form a favorable interaction. In contrast, no other contact surface, including the strand-strand dimer interface, is consistently conserved among all crystal structures (**Table 2** and **Fig. 2**).

### Mutation of W187 in the PR8 NS1 ED leads to a monomeric form

**Figure 3:**
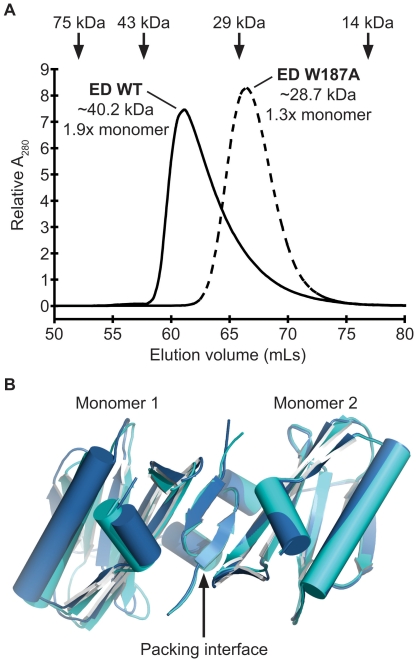
Structural analysis of PR8 NS1 ED W187A. (**A**) **PR8 NS1 ED-W187A is monomeric in solution.** Gel filtration analysis of the purified WT and W187A mutant 6His-tagged PR8 NS1 ED proteins at 1mg/mL. The elution volumes of protein standards used to calibrate the column are indicated at the top: conalbumin (75000 Da), ovalbumin (43000 Da), carbonic anhydrase (29000 Da) and ribonuclease A (13700 Da). The estimated MWs of WT and W187A PR8 EDs are shown together with their ratio to the calculated MW of the respective monomeric protein. Calibrations and calculations were performed as previously described [28]. (**B**) **Crystals of PR8 NS1 ED-W187A form by strand-strand packing.** Two crystal forms of the PR8 NS1 ED W187A monomeric mutant revealing strand-strand packing in the crystal lattice. The two monomers of each form are colored dark or light blue (PDB IDs 3O9Q and 3O9R, respectively).

While the helix-helix interface appears to be the only dimeric form of NS1 ED consistently observed in all crystal structures, it remains possible that other dimeric states (such as the strand-strand dimer) contribute to dimerization in solution. This is particularly pertinent for the PR8 NS1 ED which may be predisposed to form the strand-strand dimer in crystal lattices (e.g. PDB IDs 3O9U and 2GX9 [29]). Previous studies using recombinant Alb/76 and Ud/72 EDs have shown that a single W187A amino-acid substitution is sufficient to prevent dimerization [28], [31]. However, as the strand-strand dimer has never been observed in structures from these strains, it may be that the PR8 ED behaves differently. We therefore introduced the W187A mutation into the PR8 NS1 ED bacterial expression construct. Similar to our Alb/76 study [28], gel filtration of the purified recombinant protein confirmed that PR8 ED-W187A is likely to be monomeric as compared with the WT ED dimer, indicating that no other alternative dimer conformation (including the strand-strand dimer) forms in solution in the absence of W187 (**Fig. 3A**).

We crystallized the purified PR8 ED-W187A protein and solved the structure by molecular replacement. Two crystal forms were obtained for this protein, both occurring under the same crystallization condition but separated in time. Initially, the protein formed large hexagonal crystals belonging to spacegroup P3_2_21 (PDB ID 3O9Q). However, when the same protein preparation was used for crystallization 6 months later the protein formed long needle-shaped crystals, which belonged to spacegroup P2_1_2_1_2_1_ (PDB ID 3O9R). In both crystal forms two ED-W187A monomers were observed in the asymmetric unit (**Fig. 3B**). Surprisingly, both crystal lattices were formed through strand-strand interactions analogous to the strand-strand dimer observed in the previously published WT PR8 ED crystals (2GX9, [29]) and our 3O9U crystals. However, unlike all WT PR8 ED structures the helix-helix dimer was not present in the W187A crystal lattice. Together with our gel filtration studies, we conclude that all WT NS1 EDs predominantly form helix-helix dimers in solution. While both the WT (dimeric) and W187A (monomeric) PR8 NS1 EDs are capable of forming strand-strand dimers within the confines of a crystalline array, such dimers do not appear to exist in solution. Thus, the strand-strand interface seems to be a favorable crystallographic contact for the PR8 NS1 ED, but may not be physiologically relevant. Interestingly, this may be a strain-specific packing interface as it is not observed in any Alb/76 or Ud/72 NS1 ED structure (dimeric or monomeric) [30], [31], or even in the full-length VN/04 NS1 structure [27]. Our data cannot, however, exclude the possibility that the PR8 ED strand-strand interface is promoted during virus infection by interactions of NS1 with other proteins or by post-translational modifications.

### Consequences of breaking the NS1 ED helix-helix interface: effects on IFN-antagonism and RNA-binding

To assess functional roles of the helix-helix dimer interface in the context of full-length NS1, we introduced the W187A mutation into a PR8 NS1 mammalian expression construct. This mutation disrupts the helix-helix dimer interface resulting in a monomeric NS1 ED phenotype (**Fig. 3A**, refs. [28], [31]). Furthermore, although W187 is directly essential for Ud/72 NS1 to bind host CPSF30 (thereby contributing to efficient inhibition of cellular pre-mRNA post-transcriptional processing [33]), PR8 NS1 inherently lacks this function [2]. Thus, PR8 NS1 is the ideal NS1 to study helix-helix dimer disruption as mutation of W187 will not affect its CPSF30-binding properties, which would not be the case for NS1 proteins of many other influenza A virus strains.

**Figure 4:**
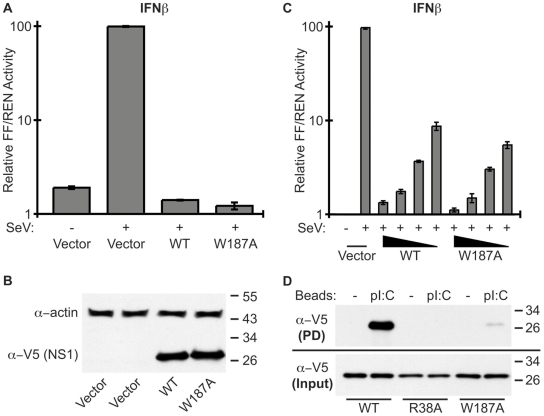
Biological role of the NS1 ED helix-helix dimer interface. **(A) Ability of the PR8 NS1-W187A mutant to antagonize IFNβ induction.** 293T cells were co-transfected for 16 h with a pCAGGS expression plasmid encoding the indicated PR8 NS1 protein (or vector only), a FF-Luc IFNβ-promoter reporter plasmid (p125Luc), and a constitutively active HSV-TK promoter driven REN-Luc reporter plasmid (pRL-TK). After infection with SeV for a further 12 h, both FF-Luc and REN-Luc activities were determined. Results represent the means and standard deviations of triplicate values (FF-Luc normalized to REN-Luc) obtained in a single experiment, and are representative of two independent experiments. Vector + SeV was set to 100%. (**B**) Western blot analysis of lysates from (**A**). NS1 and actin were detected using rabbit polyclonal antibodies. Molecular weight markers (kDa) are indicated to the right. (**C**) Assay repeated as for (**A**) but using 3.3-fold serial dilutions of pCAGGS plasmid. Total amounts of transfected DNA were kept constant. (**D**) **Ability of PR8 NS1 mutants to bind synthetic dsRNA.** Lysates from 293T cells transfected with the indicated V5-tagged PR8 NS1 construct were precipitated with pI:C-Sepharose (pI:C) or Sepharose only (−). Following SDS-PAGE, NS1-V5 proteins were detected by western blot. Molecular weight markers (kDa) are indicated to the right.

IFN-antagonism is one of the most studied functions of NS1. PR8 NS1 is thought to antagonize IFNβ promoter activation (at least in part) by binding to cellular TRIM25, thereby preventing ubiquitination of the viral RNA sensor, RIG-I [3]. The interaction of NS1 with TRIM25 requires both the NS1 RBD and ED [3], although the necessary dimeric state of the ED is unknown. We therefore assessed the ability of WT or W187A PR8 NS1 proteins to limit activation of the IFNβ promoter in 293T cells using a standard SeV-induced luciferase reporter assay. As shown in **Fig. 4A**, SeV infection induced robust amounts of IFNβ promoter-driven FF-Luc activity relative to constitutively driven REN-Luc in empty vector transfected cells (set to 100%), but not in cells expressing the WT or W187A PR8 NS1 constructs. Both NS1 constructs expressed to similar levels as determined by western blot analysis of assay lysates (**Fig. 4B**). To exclude the possibility of NS1 saturation resulting in no observable difference in IFN-antagonism, we serially-diluted the amounts of NS1 plasmid used ensuring that the total amount of transfected DNA remained constant. As shown in **Fig. 4C**, dilution of each NS1 plasmid resulted in a similar dose-response of NS1-mediated IFN-antagonism, indicating that the assays were all in the linear range. We conclude that formation of the NS1 ED helix-helix dimer (via W187) has limited impact on the ability of PR8 NS1 to inhibit IFN induction pre-transcriptionally.

Sequestration of dsRNA by the NS1 RBD has been implicated in countering several host antiviral mechanisms [3], [7], [35], although the primary consequence has been proposed to be antagonism of the 2’-5’ OAS/RNaseL pathway [7]. A previous study has implicated NS1 ED dimerization in functionally stabilizing the RBD [22]. To formally test whether ED dimerization affects the dsRNA-binding activity of NS1, we used poly I:C pull-down assays to assess the interaction of WT or W187A NS1 with synthetic dsRNA. As shown in **Fig. 4D**, WT NS1 bound efficiently to poly I:C, while no binding was observed for a dsRNA-binding incompetent mutant NS1 (R38A) [25], [36]. The W187A NS1 mutant appeared to bind poly I:C very inefficiently (**Fig. 4D**). Given that the NS1 RNA-binding site resides wholly within the RBD [37], these data suggest that ability of the NS1 ED to form helix-helix dimers is crucial for efficient RBD activity. Whether this is due to enhanced RNA-binding cooperativity of a helix-helix mediated NS1 oligomer [27], or simply the precise maintenance of the RBD dimeric structure by an ED dimer [22], is not yet clear. We speculate that formation of the NS1 ED helix-helix dimer may have biological implications for PR8 NS1 functions that require strong RNA-binding, such as antagonism of 2’-5’ OAS/RNaseL [7], but not for some other functions (e.g. potential direct binding to TRIM25 [3]).

### The helix-helix dimer interface is variable: a transient dimer?

**Figure 5:**
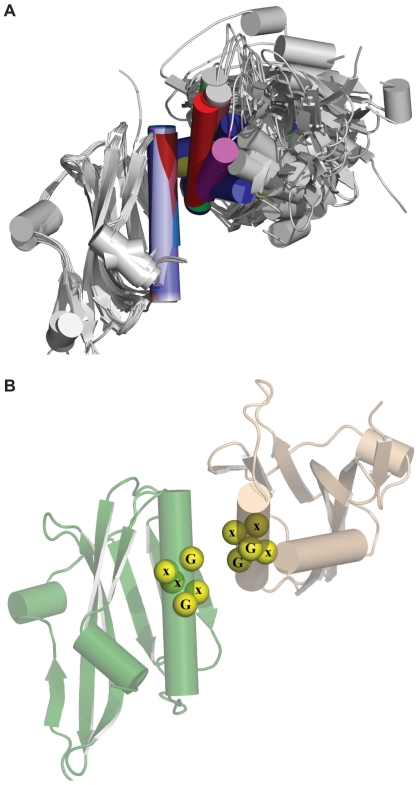
The NS1 ED forms a flexible dimer via the helix-helix interface. (**A**) Superposition of all ED helix-helix dimers oriented via the left-hand monomer of each pair to show the wide range of positions possible for the right-hand monomer, thus indicating a high degree of interface flexibility. (**B**) Highlighted GxxxG-like motif present at the helix-helix interface.

Although the helix-helix dimer interface is shared by all structures of the NS1 ED, and may have some biological role in that RNA-binding activity of NS1 is affected by its disruption, we noted that in all helix-helix structures the arrangement of the two ED monomers differs markedly relative to one another. The considerable variation in angle is not just between dimers from different strains, but is also observed between different crystal forms of the same ED (**Fig. 2** and **Fig. 5A**). This suggests an inherent flexibility to the helix-helix interaction, rather than a function of specific amino acid differences. Thus, it is possible that the two ED monomers may be able to adopt a range of orientations while still remaining in a dimeric state. Such plasticity at the monomer-monomer interface may be facilitated by the presence of several highly conserved glycine residues (G179, G183, G184) at the contact surface of the long α-helix (**Fig. 5B**). The absence of side chains at these positions allows the two monomers to come very close to one another while continuing to permit a range of orientations. These glycines may therefore constitute part of a classical GxxxG-like protein-protein interaction motif, commonly associated with α-helical homotypic binding sites [38], [39]. Such a motif is often seen in the context of transmembrane helix interactions, but has also been observed in soluble proteins such as dimers of SARS-CoV nsp9 [40].

Based on crystal structures of the NS1 ED in complex with CPSF30 and PI3K, it is clear that the helix-helix dimer is incompatible with certain NS1-host protein interactions [32], [33]. Thus, for such binding events to occur, separation of the two EDs would be required. In support of this concept, we analysed all the isolated NS1 ED structures bioinformatically and found that the helix-helix interface bears several characteristics reminiscent of transient dimers capable of associating and dissociating [41], [42], [43]: the mean interface accessible area is small (513±73 Å^2^; transient dimer <1000 Å^2^/subunit), with a small interface gap volume (3247±438 Å^3^; transient dimer <5000 Å^3^/subunit), large gap volume index (3.22±0.55 Å; transient dimer >2.0 Å), and low planarity score (RMSD = 2.53±0.14 Å^2^, transient dimer <2.5 Å^2^) (**Table 3**). An additional characteristic of transient dimers is a low local density (L_D_) of interacting atoms at the subunit interface. While obligate dimer interfaces generally have L_D_ values of around 43, transient interfaces are typified by L_D_ values in the order of 35 [44]. The ED helix-helix dimer interfaces have a mean L_D_ of 35.5±4.7 (**Table 3**). The combination of all these ‘transient’ features indicates that the helix-helix dimer is likely to be weak and thus capable of separation, potentially allowing other binding partners access to the regions buried at the interface. Similar analyses of the strand-strand dimer interface indicate that these ‘transient’ characteristics are even more pronounced (**Table 4**), confirming that this interaction is even weaker than the helix-helix dimer.

**Table 3:** Bioinformatic analysis of isolated NS1 ED helix-helix dimer characteristics.

Strain	Dimer	Symmetry relationship	Interface accessible surface area (Å^2^)*	Interface accessible surface area (%)*	Planarity (Å)*	Local density**	Gap Volume (Å^3^)*	Gap Volume Index (Å)*
PR8	2GX9_AB^HH^	asymmetric	475	6.1	2.72	31.2	2943	3.10
	3O9U_AC	asymmetric	426	6.4	2.58	27.5	3554	4.18
	3O9U_BE	asymmetric	432	6.5	2.64	28.7	3115	3.61
	3O9U_DG	asymmetric	495	7.3	2.34	35.8	3915	3.96
	3O9U_FH	asymmetric	526	8.0	2.42	35.7	3439	3.27
	3O9S_AB	asymmetric	447	6.5	2.37	31.2	2886	3.23
	3O9T_AA§	symmetric	551	8.48	2.36	37.8	2197	1.99
	3O9T_BB	symmetric	551	7.9	2.64	39.1	3854	3.50
Alb/76	3D6R_AB	asymmetric	698	10.9	2.39	39.7	3247	2.33
	3OA9_AB	asymmetric	587	9.3	2.42	39.7	2447	2.08
Ud/72	3EE8_AA	symmetric	436	6.4	2.46	30.3	2687	3.08
	3EE8_BB	symmetric	486	7.1	2.79	31.4	2754	2.83
	3EE9_AB	asymmetric	597	8.8	2.47	40.6	3459	2.90
Cal/09	3M5R_AB	asymmetric	510	6.8	2.54	40.7	3689	3.62
	3M5R_DE	asymmetric	507	7.5	2.64	39.7	3230	3.18
	3M5R_FG	asymmetric	516	7.6	2.58	39.6	3483	3.38
VN/04	3F5T_AA^ED^†	symmetric	570	8.3	2.40	38.5	4725	4.14
**Mean**			**513**	**7.5**	**2.53**	**35.5**	**3247**	**3.22**
**Standard deviation**			**73**	**1.3**	**0.14**	**4.7**	**438**	**0.55**

ED helix-helix dimers identified as PDB ID_chains (e.g. dimer 3O9U_AC is formed by chains A and C of structure 3O9U). The helix-helix dimer formed by chains A and B of PR8 ED structure 2GX9 is identified by a superscript HH to distinguish it from the strand-strand dimer also made by those chains (see Table 4). The ED dimer formed by chain A of the full-length VN/04 NS1 structure 3F5T is distinguished from the RBD dimer formed by the same chain (see Table 5) by a superscript ED. All values calculated using either ProtorP (*) or ProFace (**). Symmetric dimer 3O9T_AA (§) is excluded from calculation of mean and standard deviation due to the presence of a molecule of PEG2000 at the helix-helix dimer interface. To facilitate fair comparison, the VN/04 NS1 ED symmetric dimer 3F5T_AA^ED^(†) was not included in the calculations as it is part of a full-length NS1 structure.

**Table 4:** Bioinformatic analysis of isolated NS1 ED strand-strand dimer characteristics.

Strain	Dimer	Symmetry relationship	Interface accessible surface area (Å^2^)*	Interface accessible surface area (%)*	Planarity (Å)*	Local density**	Gap Volume (Å^3^)*	Gap Volume Index (Å)*
PR8	2GX9_AB^SS^	asymmetric	603	7.7	2.51	32.3	5812	4.82
	3O9U_AF	asymmetric	345	5.2	2.66	22.9	4357	6.31
	3O9U_BD	asymmetric	442	6.6	2.47	25.5	4165	4.71
	3O9U_CH	asymmetric	482	7.3	2.21	30.4	5461	5.67
	3O9U_EG	asymmetric	514	7.7	2.48	31.6	5883	5.73
**Mean**			**477**	**6.9**	**2.47**	**28.5**	**5135**	**5.45**
**Standard deviation**			**95**	**1.1**	**0.16**	**4.1**	**817**	**0.67**

ED strand-strand dimers identified as PDB ID_chains (e.g. dimer 3O9U_AF is formed by chains A and F of structure 3O9U). The strand-strand dimer formed by chains A and B of PR8 structure 2GX9 is identified by a superscript SS to distinguish it from the helix-helix dimer also made by those chains (see Table 3). All values calculated using either ProtorP (*) or ProFace (**).

In contrast to the ED dimer interface, the interaction between monomers of isolated apo RBDs bears more similarity to obligate than transient dimer interfaces (**Table 5**). In particular, the gap volume index is much lower (1.62±0.23 Å) and planarity much higher (3.79±0.31 Å) than observed in ED dimers. Although the interface accessible area and gap volume are still quite small (1122±60 Å^2^ and 3611±332 Å^3^, respectively) these values are likely more influenced by the size of the domain than gap volume index and planarity. However, examination of the interface surface area as a percentage of total surface area indicates that a relatively high proportion of the protein surface is involved in the interaction (21.4±0.9%, obligate dimer >20% [43]), which is in stark contrast to the ED helix-helix dimer interface (7.5±1.3%) (**Tables 3** **and** **5**). These bioinformatic analyses suggest that the RBD forms a stable obligate homodimer, while the ED helix-helix dimer (although predominant over other interfaces) may only be weak and transient.

**Table 5:** Bioinformatic analysis of isolated NS1 RBD dimer characteristics.

Strain	Dimer	Symmetry relationship	Interface accessible surface area (Å^2^)*	Interface accessible surface area (%)*	Planarity (Å)*	Local density**	Gap Volume (Å^3^)*	Gap Volume Index (Å)*
PR8	2ZKO_AB§	asymmetric	1054	19.9	3.22	35.8	3527	1.67
Ud/72	1AIL_AA	symmetric	1019	20.3	4.08	35.3	4330	2.12
Cal/09	3M8A_AG	asymmetric	1152	22.4	3.60	39.4	3419	1.48
	3M8A_BH	asymmetric	1115	21.8	3.58	38.1	3540	1.59
	3M8A_CF	asymmetric	1192	22.3	4.01	40.0	3466	1.45
	3M8A_DE	asymmetric	1115	21.4	3.39	39.1	3378	1.52
	3M8A_IL	asymmetric	1181	21.8	4.22	39.2	3679	1.56
	3M8A_JK	asymmetric	1081	20.2	3.62	39.7	3466	1.60
VN/04	3F5T_AA^RBD^†	symmetric	715	14.0	4.37	27.2	4691	3.28
**Mean**			**1122**	**21.4**	**3.79**	**38.7**	**3611**	**1.62**
**Standard deviation**			**60**	**0.9**	**0.31**	**1.6**	**332**	**0.23**

RBD dimers identified as PDB ID_chains (e.g. dimer 3M8A_AG is formed by chains A and G of structure 3M8A). RBD dimer formed by chain A of the full-length NS1 structure 3F5T distinguished from the ED dimer formed by the same chain (see Table 3) by a superscript RBD. All values calculated using either ProtorP (*) or ProFace (**). PR8 dimer 2ZKO_AB (§) is excluded from calculation of mean and standard deviation as it is in complex with a molecule of dsRNA. To facilitate fair comparison, the VN/04 NS1 RBD symmetric dimer 3F5T_AA^RBD^(†) was not included in the calculations as it is part of a full-length NS1 structure.

### Can the transient NS1 helix-helix dimer be observed in virus-infected cells?

**Figure 6:**
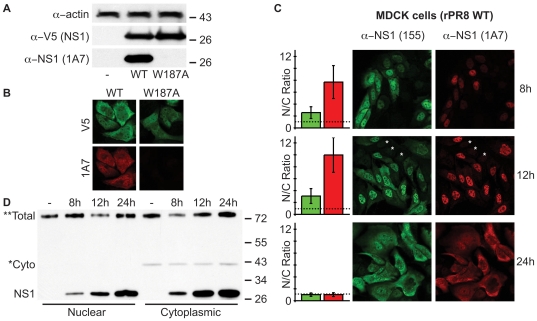
Possible visualization of ‘helix-open’ and ‘helix-closed’ NS1 ED conformational states in virus-infected cells. (**A**) **mAb 1A7 does not recognize NS1-W187A.** Western blot analysis of lysates from 293T cells transfected with pCAGGS expression plasmids encoding the indicated V5-tagged PR8 NS1 protein (or vector only). NS1 was detected using both rabbit anti-V5 pAb and mouse mAb 1A7. Actin served as a loading control. Molecular weight markers (kDa) are indicated to the right. (**B**) Indirect immunofluorescence analysis of NS1 proteins in MDCK cells transfected with pCAGGS expression plasmids encoding the indicated V5-tagged PR8 NS1 protein. Cells were fixed approximately 24 h post-transfection. Co-staining was performed using rabbit anti-V5 pAb and mouse mAb 1A7. (**C**) **mAb 1A7 and pAb 155 highlight different NS1 populations during infection.** Indirect immunofluorescence analysis of NS1 protein localization in MDCK cells infected for the indicated times with rPR8 WT virus (MOI of 2 PFU/cell). Co-staining was performed using rabbit anti-NS1 pAb 155 and mouse mAb 1A7. Asterisks indicate example uninfected cells to show background staining. Bar graphs represent the mean nuclear/cytoplasmic (N/C) ratios of mean fluorescent intensities derived from manually assigned individual nuclei and cytoplasms for each fluorescent channel (n = 25 cells per timepoint, error bars represent standard deviations). The dotted line indicates a nuclear/cytoplasmic ratio of 1. (**D**) Western blot analysis of nucleo-cytoplasmic extracts prepared from MDCK cells infected as for (**C**). The primary antibody was a polyclonal rabbit anti-serum raised against a GST-NS1 (RBD) fusion protein. *Cyto indicates a non-specific band that co-purifies solely with the cytoplasmic fractions and thus highlights purification integrity. **Total indicates a non-specific band found in all fractions that serves as a convenient loading control. Molecular weight markers (kDa) are indicated to the right.

While characterizing the functional impact of mutating W187, we serendipitously found that our V5-tagged W187A PR8 NS1 mutant construct was not recognized by the NS1-specific mAb 1A7, either by western blot on SDS-PAGs or by immunofluorescence (**Figs. 6A & 6B**). For SDS-PAGE, our cell lysis and gel loading buffer contained 6 M urea, 2 M β-mercaptoethanol and 4% SDS that, combined with boiling, should be more than sufficient to completely denature all secondary, tertiary and quaternary structures of NS1. This was clearly evidenced by its apparent mobility on the polyacrylamide gel, which corresponded to its primary sequence molecular weight (**Fig. 6A**). Thus, although we have not completely mapped the epitope of this antibody, the data strongly suggest that mAb 1A7 recognizes a linear epitope that absolutely requires W187. Given that W187 is completely buried within the ED helix-helix interface, and is not solvent exposed on this dimer surface (**Fig. 1B**), we reasoned that 1A7 would be unable to recognize the helix-helix ED dimer if this conformation actually exists. We therefore used 1A7 alongside a rabbit polyclonal NS1-specific anti-serum (pAb 155) to try and observe different sub-populations of NS1 in WT virus-infected cells, which we hypothesized may correlate with W187-exposed (‘helix-open’) or W187-buried (‘helix-closed’) conformations of NS1. MDCK cells were infected with WT rPR8 virus at an MOI of 2 PFU/cell and fixed at various times post-infection. Use of the PR8 strain prevented confounding issues regarding CPSF30 binding, which is also mediated by exposed W187 [33]. Subsequent co-staining with mAb 1A7 and pAb 155 did indeed reveal distinct NS1 populations recognized by the two antibodies (**Fig. 6C**). While pAb 155 highlighted both nuclear and cytoplasmic NS1 at 8 and 12 h post-infection, mAb 1A7 predominantly recognized only nuclear NS1 at these timepoints. However, at 24 h post-infection, both antibodies showed a similar pattern of NS1 distribution, which was divided equally between nucleus and cytoplasm. Uninfected cells within the fields-of-view acted as internal controls to ensure that observed differences were not due to differential background staining by the antibodies (**Fig. 6C**). In addition, by carefully optimizing the microscope settings for the 24 h timepoint (when NS1 levels are the highest), and not changing any conditions when switching slides or fluorescent channels, we minimized oversaturation issues. To ensure further that our image interpretations were not biased by potential differences in intensity of the two fluorescent channels of our microscope, we quantified the ratio of nuclear-to-cytoplasmic NS1 staining for both pAb 155 and mAb 1A7 at each timepoint. This ‘within-channel’ quantification totally eliminates bias, and clearly confirmed that mAb 1A7 predominantly recognizes nuclear NS1 at the 8 and 12 h timepoints, while pAb 155 recognizes a more even distribution (**Fig. 6C**). We also performed nucleo-cytoplasmic fractionation and immunoblotting of infected cells to show that total amounts of NS1 are actually slightly higher in the cytoplasm than the nucleus at all timepoints, further indicating that the observed differences are not due to relative protein abundance (**Fig. 6D**). Overall, these data suggest that at 8 and 12 h post-infection, W187 is exposed in the nucleus, while it is largely hidden in the cytoplasm, most likely by homotypic ED interactions. Given that at 24 h post-infection mAb 1A7 can recognize both nuclear and cytoplasmic NS1 in a manner similar to pAb 155 (**Fig. 6C**), we speculate that the cytoplasmic NS1 at this timepoint adopts a different conformation than the cytoplasmic NS1 at 8 and 12 h, namely that W187 is now exposed (‘helix-open’).

## Discussion

**Figure 7:**
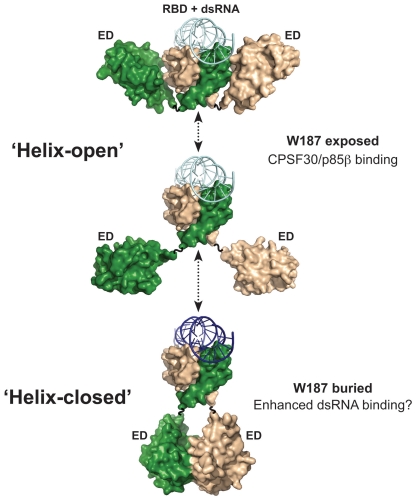
Hypothetical model of NS1 ED transient homotypic interactions. Cartoon summarizing various possible states of the NS1 ED in the context of the full-length protein. The flexible inter-domain linker permits a range of ED conformations from ‘helix-open’ to ‘helix-closed’. Such variability is promoted by the helix-helix interface being weak and transient. The mAb 1A7 only recognizes NS1 when W187 is exposed (‘helix-open’). Some functions potentially associated with each conformer are listed. It is likely that certain states are temporally or spatially regulated during infection, either by protein-protein interactions or by post-translational modifications. The NS1 monomers are colored green and wheat. The dsRNA is colored in shades of blue to represent possible different binding affinities to NS1. Models generated using the following PDB IDs: 3F5T, 2RHK, and 2ZKO.

Although much is known about the plethora of molecular interactions between influenza A virus NS1 protein and cellular factors [1], surprisingly few studies have addressed potential mechanisms by which NS1 multifunctionality is achieved. Present models of the tertiary and quaternary structures of NS1 indicate that the protein possesses two globular domains, each capable of forming independent homodimeric interactions. Our bioinformatic analyses indicate that the N-terminal RBD forms a very stable homodimer, while the predominant and ubiquitous helix-helix contacts between C-terminal EDs bear many similarities with weak transient interfaces. Thus, while the RBD dimer is likely to remain constant, the ED may move between monomeric and dimeric states. We therefore propose a new model of NS1 functional regulation dependent upon several different molecular arrangements of the protein, which may be temporally, spatially, interaction, or modification induced. Our model is summarized in **Fig. 7**.

By integrating structural and biochemical data, we have very tentatively identified at least two NS1 conformations in virus-infected cells, which we have termed ‘helix-open’ (monomeric ED conformation) and ‘helix-closed’ (dimeric ED helix-helix conformation, or shielding by another cellular/viral protein). The exact biological function of these possible NS1 conformational states has yet to be fully defined, yet some information is already available. For example, our studies using a mAb that should not recognize the ‘helix-closed’ conformation suggest that the two conformations may have specific spatio-temporal distributions during virus infection. Furthermore, the rationally-designed ‘helix-open’ NS1 protein (W187A) was poor at binding synthetic dsRNA, perhaps indicating that ED helix-helix dimeric interactions allosterically increase affinity of the RBD for dsRNA, or that ED homotypic interactions facilitate oligomerization of NS1 and thus cooperative binding to dsRNA. In this regard, it is worth noting that a previous study proposed a multimeric state of NS1 composed of extensive repeating chains of ‘domain-swapped dimers’ [27]. The chains were noted in a crystal lattice of full-length VN/04 NS1, and may explain the long NS1 filaments formed at high protein concentrations [27]. The precise role, if any, of such filaments is unknown, although NS1 ‘tubules’ made up of multiple filaments were proposed to be involved in cooperatively binding dsRNA [27]. Our data may therefore be consistent with such a general multimerization model, although it is clear from both previous studies [23], and the actual structure of dsRNA in complex with the RBD [25], that NS1 ‘tubules’ are incompatible with dsRNA binding, as dsRNA would be positioned perpendicular to the chain of NS1 molecules. Thus, any dsRNA binding cooperativity exhibited by NS1 ED helix-helix dimerization or multimerization is likely to be independent of the ‘tubules’ seen in the VN/04 crystal lattice [27].

Evidence from the published crystal structures of NS1 ED in complex with domains from cellular proteins also confirms that the ED helix-helix interface must be able to separate [32], [33]. The NS1 proteins of most influenza A virus strains bind the nuclear-localized 30 kDa subunit of cleavage and polyadenylation specificity factor (CPSF30) as a mechanism to suppress general host gene expression [2], [4], [13], [15]. The CPSF30 binding site on NS1 overlaps extensively with the ED helix-helix dimer interface [28], [33], indicating that the NS1:CPSF30 complex and the NS1 ‘helix-closed’ conformations are mutually exclusive. It is highly likely that the affinity of NS1 for CPSF30 is much higher than that of its homotypic interaction, at least in the absence of any other stabilizing factors. The ability of NS1 to only interact with CPSF30 when in the ‘helix-open’ state is a prediction consistent with our observation that this NS1 conformation is predominantly found in the nucleus at early times post-infection, when suppression of host-cell gene expression may be most critical. NS1 also activates host cytoplasmic PI3K signaling at mid-times post-infection by directly binding the p85β regulatory subunit of this enzyme [9]. We recently reported the crystal structure of this interaction [32], and while the ‘helix-closed’ conformation of NS1 does not preclude binding to p85β, our careful modelling of the complete heterotrimeric NS1:PI3K complex suggested that the ‘helix-closed’ NS1 state was unlikely to be relevant in a biological context due to steric clashes with the membrane upon which active PI3K must be associated. Thus, binding of NS1 to either CPSF30 or PI3K would require ‘helix-open’ conformations. Although we apparently only detect a small proportion of ‘helix-open’ NS1 in the cytoplasm relative to nucleus at 8 h post-infection, this may be explained by the fact that NS1-mediated activation of PI3K is a catalytic process, thus requiring few NS1 molecules, whereas CPSF30 inhibition requires stoichiometric sequestration by many NS1 molecules. In addition, the kinetics of NS1 performing its many functions are far from clear, and several other factors beyond those discussed here are likely to affect the conformational state of NS1. Indeed, in our model, we envisage that certain cellular interactions or post-translational modifications, such as phosphorylation [10] or SUMOylation [12], may temporally and spatially regulate NS1 conformations.

A key aspect of our functional model is the instability and flexibility of the NS1 ED helix-helix dimer, which seems weak and transient *in vitro*, but may be slightly more stable in the context of the full-length protein, or become more permanent in the presence of certain binding partners. ED dimer flexibility is likely permitted by the presence of several glycine residues at the homodimer interface (a putative GxxxG protein-protein interaction motif) that allow the two helices to rotate easily relative to one another. Mutation of one of these glycines to a charged bulky residue (G184R) has recently been reported to decrease efficient virus replication and virulence in a mouse model without affecting tissue-culture replication or the IFN-antagonistic properties of NS1 [45]. This substitution is highly likely to have altered the flexibility between, or self-association of, two homotypic EDs. Interestingly, previous gross truncation of the NS1 ED to prevent dimerization also had minimal impact on virus replication in tissue-culture, but substantially reduced virulence in mice, a property largely reversed by artificially grafting a heterologous multimerization motif onto the truncated NS1 protein [22].

Many functions of NS1 are strain-specific [2], [5], [13], [14], [15], [17], [18], and there is evidence to suggest that NS1 ED dimerization may also vary between influenza A virus strains. In particular, a previous report found that the Ud/72 NS1 ED requires concentrations in excess of 0.3 mM (approximately 5 mg/mL) for dimerization to occur [31]. Our preliminary results also indicate that at 0.06 mM (approximately 1 mg/mL) the purified Ud/72 NS1 ED is predominantly monomeric (*data not shown*). However, at this same concentration we routinely observe complete dimerization of the PR8 and Alb/76 NS1 EDs. It is tempting to speculate that ED dimerization affinity may contribute to some of the strain-specific activities that NS1 performs, particularly if the NS1 ED of some strains is more likely than others to self-associate.

Multiple NS1 ED positions are likely facilitated by the short flexible inter-domain linker that exists between the RBD and ED (usually residues 74*–*86). Presence of this linker suggests that the RBD and ED are never spatially fixed relative to one another, with independent dimerization of each domain occurring. Intriguingly, inter-domain linker length can also vary between influenza A virus strains, and evidence suggests that a 5 amino-acid deletion in this region significantly impacts upon virulence [18]. The exact mechanism by which this is achieved is not well understood, although we postulate that shorter linkers may restrict the range of positions potentially adopted by the NS1 ED.

Weak transient protein:protein interactions that readily associate and disassociate are an important regulatory mechanism adopted by several multifunctional cellular proteins [46]. Here, we have used structural, bioinformatic, and biological evidence to propose a similar mode of action for the influenza A virus NS1 protein, an important virulence factor. Our new hypothetical model may help explain how such a small protein has been able to expand its number of cellular and viral binding partners. Further work to accurately define the existence and potential regulation of NS1 conformers in solution, both structurally and during infection, will be essential for understanding some of the complexities of this remarkable protein.

## Materials and Methods

### Protein expression and purification

The pRSFDuet-1 plasmid expressing a 6His-tagged version of the Alb/76 NS1 ED (residues 73–230) has been described previously [28]. The same cloning strategy was used to generate a plasmid for expression of a 6His-tagged version of the PR8 NS1 ED (residues 73–230). Four-primer overlap PCR was used to specifically introduce nucleotide changes encoding the W187A mutation into the PR8 NS1 ED cDNA. The identity of each construct was confirmed by commercial DNA sequencing. Recombinant 6His-tagged NS1 EDs were expressed in E. coli strain BL-21 (DE3), purified, 6His-tag removed, and gel filtrated as described previously [28].

### Crystallization, data collection and structure solution/refinement

Crystals were obtained by vapor diffusion in hanging drops consisting of 2 µL concentrated protein solution (approx 8–10 mg/mL in 50 mM Tris-HCl [pH 7.8], 0.2 M NaCl) and 2 µL of reservoir solution. Reservoir solutions for each structure were as follows (identified by PDB ID): 3O9S (12% PEG 3350, 0.05 M NaSCN, 0.1 M Bis-Tris [pH 5.5], 15% glycerol, 0.05 M DTT); 3O9T (0.1 M Tris-HCl [pH 6.5], 0.1 M NaCl, 20% PEG 4K); 3O9U (0.1 M Tris-HCl [pH 6.5], 0.2 M NaCl, 25% PEG 6K); 3OA9 (0.2 M sodium formate, 20% PEG 3350). For both 3O9Q and 3O9R the reservoir solution was 0.1 M HEPES [pH 7.0], 0.2 M NaCl, 22% PEG 6K, 10% isopropanol, 10% glycerol.

Data were collected on an in-house rotating anode (RA Micro7 HFM) and a Saturn944 CCD at 100K and processed with HKL2000 [47]. Structures were solved by molecular replacement using PHASER [48] and refined using Refmac5 [49] and PHENIX [50] with manual model building using O [51] and Coot [52]. Figures were created using PyMol [53].

### Bioinformatic Analyses

Domain interface characterization was performed using a dataset comprised of both novel NS1 structures and coordinates from the PDB database. Structures of full-length NS1 or domains in complex with other molecules (e.g. dsRNA or p85β) were excluded from the dataset. Properties of each interface were calculated using ProtorP [54] and ProFace [55].

### Mammalian cells and antibodies

293T and MDCK cells were purchased from the American Type Culture Collection (ATCC, VA, USA) and maintained in DMEM supplemented with 10% FBS, 100 units/mL of penicillin, and 100 µg/mL of streptomycin (GIBCO Life Technologies, CA, USA). Mouse and rabbit anti-V5 antibodies were purchased from Serotec (UK), and rabbit anti-actin was from Sigma-Aldrich (MO, USA). The rabbit polyclonal NS1 anti-serum (pAb 155) was kindly provided by Peter Palese (Mount Sinai School of Medicine, USA). The rabbit polyclonal NS1 anti-serum raised against a GST-NS1 (RBD) fusion protein was used previously [15]. The anti-NS1 hybridoma (mAb clone 1A7) was a generous gift from Jonathan Yewdell (Laboratory of Viral Diseases, NIAID, NIH, USA).

### Plasmids

A mammalian expression construct for C-terminally V5-tagged PR8 NS1 under control of the chicken β-actin promoter was generated by amplifying the entire PR8 NS1-V5 ORF from an existing clone [9] and ligating it into the EcoRI and XhoI restriction sites of pCAGGS [56]. As required, four-primer overlap PCR was used to introduce site-directed point mutations into the NS1-encoding cDNA. All NS1-encoding cDNAs also contained silent mutations in the splice acceptor site in order to prevent expression of NS2/NEP [9]. The reporter plasmid with the firefly luciferase (FF-Luc) gene under control of the IFNβ promoter (p125Luc) was kindly provided by Takashi Fujita (Kyoto University, Japan) [57]. The reporter plasmid with the *Renilla* luciferase gene (REN-Luc) under control of the constitutively active HSV-TK promoter (pRL-TK) was purchased from Promega, WI, USA. Identity of each new construct was confirmed by commercial DNA sequencing.

### Viruses

Stocks of Sendai virus (SeV; Cantell strain) propagated in 10-day old embryonated chicken eggs were kindly provided by Silke Stertz (Mount Sinai School of Medicine, USA). Recombinant rPR8 (wild-type, WT) was rescued according to a previously reported protocol [15], albeit using PR8 RNA expression plasmids. The plaque-purified rescued virus was propagated and titrated in MDCK cells, and the genotype confirmed by RT-PCR and sequencing of the entire NS segment.

### IFNβ reporter assays

For analysis of IFNβ promoter activation, 293T cells in 12-well plates were transfected with 25 ng of p125Luc, 25 ng of pRL-TK, and the indicated amount of PR8 NS1-V5 (or empty vector) expression plasmid using FuGENE6 (Roche, WI, USA). After 16 h, the cells were infected with approximately 1 PFU/cell of SeV for 12 h. Cells were harvested and lysed in 200 µL of passive lysis buffer (Promega, WI, USA), and both FF-Luc and REN-Luc activities were determined using the Dual-Luciferase® reporter assay system as directed by the manufacturer (Promega, WI, USA). All transfections were carried out in triplicate, and experiments were independently repeated twice.

### Poly(rI):poly(rC)-Sepharose pull-downs, SDS-PAGE and western blot

Poly(rI):poly(rC) (pI:C) Sepharose pull-downs were performed as described [15], albeit using transiently-expressed (293T) PR8 NS1-V5 proteins as bait. Nucleo-cytoplasmic extracts were prepared using commercial nuclear and cytoplasmic extraction reagents (NE-PER®, Thermo Scientific, IL, USA). For western blot analysis, lysates were prepared in disruption buffer (6 M urea, 2 M β-mercaptoethanol, 4% SDS), sonicated to shear nucleic acids, and boiled for 5 min prior to polypeptide separation by SDS-PAGE on 4–15% Tris-HCl gradient gels (Bio-Rad Laboratories, CA, USA). Proteins were detected by standard western blot techniques following transfer to polyvinylidene difluoride (PVDF) membranes.

### Immunofluorescence studies

For indirect immunofluorescence staining, MDCK cells were seeded onto glass-bottomed 12-well culture plates and transfected (1 µg plasmid per well) or infected (2 PFU/cell) as described above. At the indicated times, cells were fixed and permeabilized with ice-cold methanol for 5 min at 4°C and blocked for 1 h in PBS containing 1% bovine serum albumin (BSA). Primary antibodies (see above) were incubated with the samples for 1 h at room temperature. Secondary antibodies (goat anti-mouse Alexa Fluor 633 or goat anti-rabbit Alexa Fluor 488, Invitrogen, USA) were incubated with the samples at a 1∶1000 dilution prior to fluorescence imaging using a Zeiss LSM 510 Meta confocal microscope. Mean fluorescence intensities of manually selected nuclei and cytoplasms were quantified using ImageJ [58].
